# Impacts of African Swine Fever on Pigmeat Markets in Europe

**DOI:** 10.3389/fvets.2020.00634

**Published:** 2020-09-11

**Authors:** Jarkko K. Niemi

**Affiliations:** Bioeconomy and Environment Unit, Natural Resources Institute Finland (Luke), Seinäjoki, Finland

**Keywords:** African swine fever, trade, pigmeat, supply, markets, producer price, export

## Abstract

African Swine Fever (ASF) is a highly contagious animal disease which can cause disruptions in the international trade of pigs and products derived from pigs. During 2014–2019 ASF was introduced into several member states in the European Union (EU), including the Baltic states and Poland (2014), Czech Republic and Romania (2017), Belgium, Bulgaria and Hungary (2018), and Slovakia (2019). The objective of this study was to analyze how the ASF epidemic has contributed to the production, export, and prices of pigmeat and to the national pig inventory (number of pigs) in 11 EU member states. The data included country-level statistics on the pig markets and ASF outbreaks observed during 2010–2019. The data were first analyzed descriptively. Following this, a system of four equations was specified and estimated by using seemingly unrelated regression method. The results indicated that the consequences of ASF to the pigmeat markets are complex and may differ by country. They suggest that an ASF outbreak can reduce the production of pigmeat, export quantities and the national pig inventory in the short and medium term. On average, those new cases of ASF reduced the exports of pigmeat by close to 15% and the production quantity by more than 4% in the year after the cases had occurred, and the national pig inventory by 3–4% both in the current and the next year. However, only indirect effects on pigmeat prices were observed.

## Introduction

African swine fever (ASF) is a notifiable contagious animal disease, the control of which is governed by national and international regulations and agreements. Apart from the island of Sardinia in Italy, the European Union (EU) was free from ASF for many years until the disease was introduced into Lithuania in 2014. However, the disease was introduced into non-EU countries in Eastern Europe already a few years earlier, namely into Georgia (in April 2007), the Russian Federation (2007), Ukraine (2012), and Belarus (2013). Since 2014, the disease has spread to nine Eastern European member states of the EU [see (1, 2) for an overview of how the situation has evolved over time]. In some member states, such as Poland, the disease was limited to a small part of the country and not to the entire country (3). Because of the emergence of ASF in Eastern Europe, ASF is considered to pose a risk to both Eastern and Western European countries (4). In particular, as a route of spreading the disease, the wild boar has been of concern (5).

The member states and farming sector are concerned about the economic consequences caused by possible ASF outbreaks, measures to control and eradicate the disease, and market implications of the disease. The EU has adopted a common policy to control ASF. This policy includes measures such as the culling of susceptible animals, cleaning and disinfecting infected premises, and imposing restrictions on pig transports in surveillance and protection zones which are established around the infected premises (6). The restrictions on intra-community trade are however imposed on regional basis, which implies that not the entire country may face restrictions to trade within the EU when ASF is detected in the country (7). In addition, the European Commission may adopt acts taking exceptional support measures (such as financial support) for the affected market in order to take account of restrictions on trade as a result from the application of measures for combating the spread of diseases in animals (8), which implies that the market effects may be limited by support policies.

Scientific literature suggests that ASF outbreaks, even if they are small, can cause substantial economic losses to pig farming in the affected states [e.g., (9, 10)]. Because ASF poses a sanitary risk, countries have the right to prohibit imports of pigs and products of pig origin from the areas where ASF is or has been present (11). The impact of ASF on the international trade of pigs and products of pig origin is of particular interest, and ASF has been argued to reduce export quantity and the price of pigmeat in the country where it has been detected [e.g., (9, 10)]. In addition to animal health considerations, the effects that trade restrictions caused by ASF may have on exports of pig products and on producer prices in the domestic markets are a major reason why stakeholders are concerned about the risk of disease in the EU. However, while a highly contagious animal disease has the potential to cause substantial economic damage, the impacts of the disease can vary from country to country, and the characteristics of a country, such as export orientation and the level of development of the industry, may contribute to the impacts (12).

Especially in developed countries, economic impacts of a highly contagious animal disease are often studied ex-ante by using simulation models whereas ex-post studies are less common. There are a few examples where the market implications of a highly contagious animal disease have been studied by using econometric methods and time series data. For instance, Jarvis et al. (13) investigated beef prices in different markets and observed that foot and mouth disease (FMD) free producers enjoyed a higher price than producers from FMD-endemic countries. In addition, Wilson and Kinsella (14) studied the impact of FMD on the price of beef in the United Kingdom following the 2001 epidemic, and Barratt et al. (15) used a time series analysis to estimate the indirect costs of animal disease control strategies using a FMD outbreak in Scotland as a case study. There are several studies which have looked at the financial or economic impacts of other animal diseases ex-post. Such diseases include for instance porcine reproductive and respiratory syndrome (16), bovine spongiform encephalopathy (17), and bluetongue (18).

Although the economic consequences of ASF outbreaks and their control in Europe have been addressed in various countries [e.g., (10, 19–21)], the impact of the ASF epidemic in the Eastern European pigmeat market have not, according to the author's knowledge, been investigated retrospectively and therefore long-term impacts have not been verified. The epidemic that started in the EU in 2014 provides a good opportunity to quantify the effects of ASF on markets at country level, and the information could be utilized when considering policies and support measures to the pig farming sector. Hence, the objective of this study was to analyze how the ASF epidemic has contributed to the production, export, national pig inventory and prices of pigmeat in the EU member states where it was observed during the period 2010–2019.

## Materials and Methods

### Model

The analysis included two steps. First, a descriptive analysis was conducted. The evolution of the price of pigmeat, annual production and export quantities of pigmeat, and the national pig inventory (the number of domestic pigs in the country) for a time period of 10 years were examined by using empirical data described in the next section. The period was selected so that it included several disease-free years for all countries expect Italy and provided information on ASF until the most recent year for which data were available. In addition, Pearson correlation coefficients were computed and presented to carry out a preliminary analysis of raw data.

Second, a set of four equations describing the effects of the number of infected wild boars, the number of ASF infected domestic pig farms, lagged values of market parameters and dummy variables to year-to-year change in the price of pigmeat, the annual production and export quantities of pigmeat, and the national pig inventory in the country were specified in a reduced form and estimated. Because of serial correlation issues, the dependent variables of equations were in first-differenced form as follows.

$$\Delta x_{i,t}=\alpha_{i}+\beta_{i}x_{t-1}+\delta_{i}y_{t}+\theta_{i}z_{t}+\varepsilon_{i,t}$$

for *i* = {Price of pigmeat, Production quantity, Export quantity, Pig inventory} and where Δ*x*_*i,t*_ = *x*_*i,t*_−*x*_*i, t*−1_; where *t* is the time index; Δ*x*_*i,t*_ represents the change in the natural logarithm (*ln*) of the dependent variable *i* from time *t–*1 to time *t*; *i* is the variable name index; *x*_*i,t*_ represents the variable *i* (price of pigmeat, production quantity, export quantity or pig inventory) at the time period *t*; α_*i*_ is the intercept; ****β****_**i**_, ****δ****_**i**_, and ****θ****_**i**_ are vectors of estimated parameters; **x**_**t−1**_ represents a vector of *ln*-transformed variables price of pigmeat, production quantity, export quantity and pig inventory in period *t–*1; **y**_**t**_ is a vector of dummy variables representing the year and country of observation; **z**_**t**_ is a vector representing six variables [the dummy variable that ASF has been observed in wild boar in the country, dummy variable that ASF has been observed in domestic pigs in the country, *ln*(1 + number of new ASF positive pig farms in year *t*), *ln*(1 + number of new ASF positive pig farms in year *t–*1), *ln*(1+number of new ASF infected wild boars in year *t*), *ln*(1+number of new ASF infected wild boars in year *t*-1)]; and ε_*i,t*_ is an error term for the equation representing variable *i*.

The system of simultaneous equations was estimated by using seemingly unrelated regression equations (SURE) method (22). This method is suited for estimating equations which have a specific form of the variance-covariance matrix, i.e., equations in cases where the error terms of estimating equations are correlated. This can be the case when variables are determined simultaneously. For instance, the supply, demand and price of a product are likely to be determined simultaneously and therefore the error terms of equations representing these can be correlated. The problem can be taken into account by using the SURE method [see, e.g., (23)].

The system of four equations was estimated in a single iterative model run. Due to the model structure explained above, country-specific levels were considered as random effects whereas country-specific trend and year-specific effects were considered as fixed effects. Annual dummy variables also implicitly included the effects of events such as ASF outbreak in Asia. The estimation procedure was initiated by including all explanatory variables in each of the four simultaneous estimation equations. However, in the final model only variables which were statistically significant at a risk level of 5% were included. The variables were excluded from the model stepwise by dropping the least significant variable (*p* > 0.05) from each equation after each estimation round, and then re-estimating the system of equations until all the variables remaining in the model were statistically significant at a risk level of 5%. The estimations were conducted with an econometrics toolbox (24) in Matlab R2014b (The MathWorks Inc., Natick, Massachusetts).

### Data

The data included annual market information on producer prices, production, and volume of export of pigmeat as well as the number of pigs (the national pig inventory) in ten EU member states where ASF had been reported between 2010 and 2019 (Belgium, Bulgaria, the Czech Republic, Estonia, Italy, Latvia, Lithuania, Hungary, Poland, Romania, and Slovakia). In addition, data for Germany and Denmark were illustrated in the descriptive analysis to provide information from major pig producing countries, which did not have an ASF outbreak during the study period. The data were obtained from publicly available statistics and records. Information on the number of ASF cases detected in each country in each year was retrieved from the European Commission Animal Disease Notification System (25). While ASF has been endemic in the island of Sardinia in Italy, in other countries it was introduced during 2014 through to 2019. The largest number of ASF cases had been observed in Poland, Romania, Italy and the Baltic countries (Table 1).

**Table 1:** Number of outbreaks of with ASF in domestic pigs and in wild boar per country and per year in the European Union during years 2010–2019.

**Year**	**Belgium**	**Bulgaria**	**Czech Republic**	**Estonia**	**Italy**	**Latvia**	**Lithuania**	**Hungary**	**Poland**	**Romania**	**Slovakia**
**Number of outbreaks in domestic pigs**
2010	0	0	0	0	9	0	0	0	0	0	0
2011	0	0	0	0	31	0	0	0	0	0	0
2012	0	0	0	0	74	0	0	0	0	0	0
2013	0	0	0	0	109	0	0	0	0	0	0
2014	0	0	0	0	40	32	6	0	2	0	0
2015	0	0	0	18	16	10	13	0	1	0	0
2016	0	0	0	6	23	3	19	0	20	0	0
2017	0	0	0	3	17	8	30	0	81	2	0
2018	0	1	0	0	10	10	51	0	109	1,163	0
2019	0	44	0	0	1	1	19	0	48	1,724	11
**Number of outbreaks in wild boar**
2010	0	0	0	0	1	0	0	0	0	0	0
2011	0	0	0	0	3	0	0	0	0	0	0
2012	0	0	0	0	17	0	0	0	0	0	0
2013	0	0	0	0	67	0	0	0	0	0	0
2014	0	0	0	41	70	148	45	0	30	0	0
2015	0	0	0	723	46	753	111	0	53	0	0
2016	0	0	0	1,052	132	865	303	0	80	0	0
2017	0	0	202	637	93	947	1,328	0	741	0	0
2018	161	5	28	230	64	605	1,443	138	2,438	170	0
2019	482	165	0	80	60	369	464	1,598	2,468	683	27

*Source: European Commission, Animal Disease Notification System (ADNS)^a^*.

^a^*An “outbreak” means the holding or place situated in the territory of the Community where animals are assembled and where one or more cases of ASF has or have been officially confirmed. For instance, in Estonia the number of reported cases of ASF in wild boar (26) has been larger than the number of outbreaks of ASF in wild boar*.

The annual prices of pigmeat (class E) were retrieved from the European Commission (27). The export quantities of pigmeat, the quantity of pigmeat produced and the pig inventory (all domestic pigs in the country), were obtained from the Eurostat database (28). These exports included all fresh, frozen, cured, smoked and other pigmeat, and other products specified as pig product in the combined nomenclature CN8 categories starting with CN02 or CN15; but it did not include preparations which contained other meat besides pigmeat. Quantitative variables were converted to an index so that the base year for each country was 2010 (=100), the purpose of which was to reduce the scale effect in some cases. However, the characteristics of the countries were taken into account by the inclusion of country-specific dummy variables. For estimation purposes, continuous variables were ln-transformed. Means and the standard deviations of variables used in the seemingly unrelated regression equations estimation are presented in Table 2.

**Table 2:** Mean and standard deviation of parameters used in the seemingly unrelated regression equations estimation.

**Variable**	**Mean**	**Standard**
		**deviation**
Change in ln(Production quantity of pigmeat) from year *t*−1 to year *t*	0.019	0.086
Change in *ln*(Price of pigmeat) from year *t–*1 to year *t*	0.020	0.109
Change in *ln*(National pig inventory) from year *t–*1 to year *t*	−0.025	0.065
Change in *ln*(Export quantity index of pigmeat) from year *t–*1 to year *t*	0.036	0.278
Intercept	1.000	0.000
Dummy variable, 1 if year 2012; otherwise 0	0.111	0.316
Dummy variable, 1 if year 2013; otherwise 0	0.111	0.316
Dummy variable, 1 if year 2014; otherwise 0	0.111	0.316
Dummy variable, 1 if year 2015; otherwise 0	0.111	0.316
Dummy variable, 1 if year 2016; otherwise 0	0.111	0.316
Dummy variable, 1 if year 2017; otherwise 0	0.111	0.316
Dummy variable, 1 if year 2018; otherwise 0	0.111	0.316
Dummy variable, 1 if year 2019; otherwise 0	0.111	0.316
Dummy variable, 1 for Bulgaria, otherwise 0	0.091	0.289
Dummy variable, 1 for the Czech Republic, otherwise 0	0.091	0.289
Dummy variable, 1 for Estonia, otherwise 0	0.091	0.289
Dummy variable, 1 for Italy, otherwise 0	0.091	0.289
Dummy variable, 1 for Latvia, otherwise 0	0.091	0.289
Dummy variable, 1 for Lithuania, otherwise 0	0.091	0.289
Dummy variable, 1 for Hungary, otherwise 0	0.091	0.289
Dummy variable, 1 for Poland, otherwise 0	0.091	0.289
Dummy variable, 1 for Romania, otherwise 0	0.091	0.289
Dummy variable, 1 for Slovakia, otherwise 0	0.091	0.289
Dummy variable, 1 if ASF reported in the country in *t*, otherwise 0	0.455	0.501
*ln*(Price of pigmeat, year *t–*1)	5.045	0.120
*ln*(Production quantity index for pigmeat, year *t–*1)	4.662	0.230
*ln*(National pig inventory, year *t–*1)	4.484	0.111
*ln*(Quantity index for exported pigmeat, year *t–*1)	4.910	0.488
*ln*(1+number of new ASF positive pig farms, year *t–*1)	0.866	1.518
*ln*(1+number of new ASF cases in wild boat, year *t–*1)	1.7408	2.5726
*ln*(1+number of new ASF positive pig farms, year *t*)	1.0649	1.7016
*ln*(1+number of new ASF cases in wild boat, year *t*)	2.309	2.790

## Results

### Descriptive Analysis

**Figure 1:**
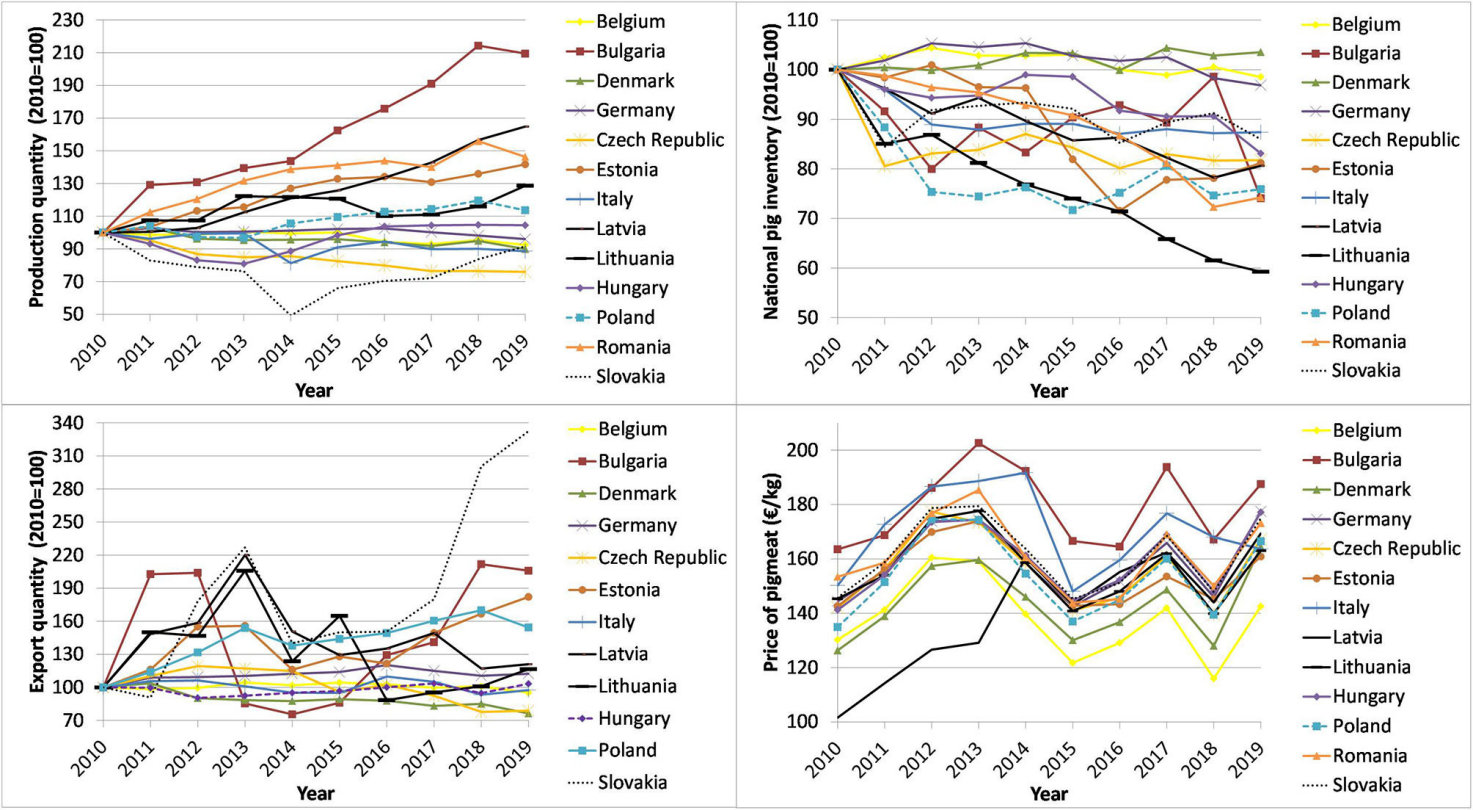
The evolution of the quantity of pigmeat produced, the national pig inventory, the quantity of exported pigmeat (2010 = 100), and the price of pigmeat (class E, €/kg) in the study countries and in Germany and Denmark. Romania was not included in the export graph because of visualization reasons. Sources: Eurostat, the European Commission.

The data indicated that the pig sector has evolved differently in countries where ASF has been detected during the past few years (Figure 1). In some countries, such as Bulgaria and Latvia, there has been a clear increasing trend in the production quantity while in some other countries, such as the Czech Republic, the production has decreased during the past decade. The national pig inventory in general had decreased in all ten countries, which is likely associated with the increased productivity of pig farming. There was a clear decrease in the national pig inventory especially in Lithuania. The development of producer price of pigmeat and the quantity of exported pigmeat over the decade varied from year to year in most countries. The quantity of exports from Romania was not included in the Figure because of large differences between the years. The quantity of pigmeat exported from Romania increased up to index value 426 by year 2012 (2010 = 100) and further to the value 883 in 2017, but thereafter the export index decreased to 553 in 2018 and to 179 in 2019. However, the initial amount of pigmeat exported from Romania was low [for further information on pig sector in Romania, please see Popescu (29)]. In addition, Poland had increased exports at the end of the decade when compared to year 2014 (Figure 1).

When considering the changes from 2013 to 2015, i.e., from the year before the major ASF epidemic started in Eastern Europe until the second year of the epidemic in each country where ASF had been observed in 2014, the national pig inventory decreased in all four countries where ASF was introduced (Estonia −15.1%, Latvia −9.1%, Lithuania −8.9%, Poland −3.7%). These changes were larger and more negative than in most other countries in the data for the same period. In these other countries, the change (from 2013 to 2015) ranged from +4.0% in Hungary to −0.6% in Slovakia, and Romania was an outlier in this group of countries with a change of −4.9%. Changes observed in the pig inventory between 2017 and 2019, when ASF was introduced into Czech Republic, Romania, Belgium, Bulgaria, Hungary, and Slovakia, varied by country.

**Figure 2:**
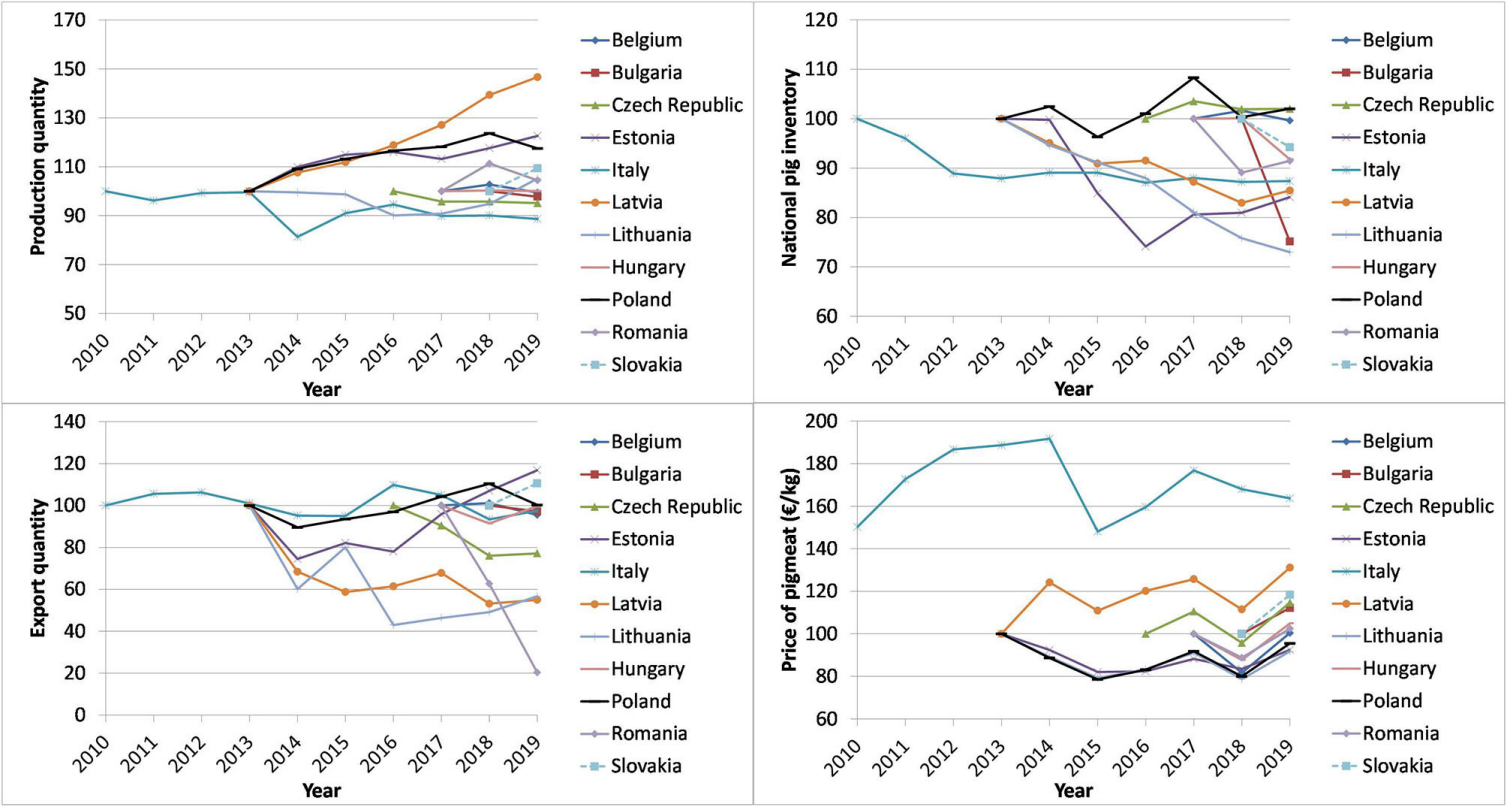
The evolution of the quantity of pigmeat produced, the national pig inventory, the quantity of exported pigmeat (value in the year before ASF was observed in the country = 100), and the price of pigmeat (class E, €/kg) in the study countries starting from the year before ASF was observed in each country. Sources: Eurostat, the European Commission.

From 2013 to 2015, a clear increase in the quantity of produced pigmeat was observed in three of four countries where ASF had been introduced in 2014 (Estonia +14.9%, Latvia +11.9%, Poland +13.2%). Also three countries where ASF had not occurred, showed an increase in production volumes during the same period (Bulgaria +16.6%, Hungary +21.6%, Romania +7.1%). The remaining countries (Belgium, Czech Republic, Italy, Lithuania) witnessed a decrease in production quantity. However, in a descriptive analysis it remained unclear what the contribution of ASF to these changes was. The quantity of exported pigmeat varied from year to year and country to country. From 2013 to 2015, Estonia (−17.9%), Latvia (−41.3%), Lithuania (−19.9%) and Poland (−6.5%) all faced a decrease in the quantity of exported pigmeat. In these countries ASF had been introduced in 2014. However, four other countries had also faced a decrease (ranging from −0.1 to −34%) in pigmeat exports during the same time period. Of those countries where ASF had been introduced in 2017 or 2018, the export quantities in the year after the introduction of ASF into the country were lower than export quantities in the year before the introduction of ASF (Belgium −1.6%, the Czech Republic −24.0%, Hungary −0.3%, Romania −79.7%). In other countries, the changes in the exports during the same period were in the range of −18.9 to +37.1% (Figure 2).

Overall, the price of pigmeat evolved quite similarly during the study period in all other countries except in Latvia, where the price development of class E pigmeat deviated from the generic EU price development after 2013 and increased overall by 67% during the decade (Figure 1). During 2013–2015, the producer price of class E pigmeat decreased by 17.8–23.5% in all other countries except Latvia, where the price increased by 10.9%.

Table 3 shows Pearson correlation coefficients for indices representing production and export quantities and the number of pigs, the price of pigmeat and the number of ASF cases in the country. Most correlations were statistically significant and it is likely that also the equations representing the evolution of these parameters have correlated error terms. The price of pigmeat correlated negatively with all other parameters. Statistically significant correlations between parameters other than the price of pigmeat were positive.

**Table 3:** Pearson correlation coefficients (upper triangle, in roman style) and their *p*-values (lower triangle, in italics) of market parameters and the number of ASF outbreaks in the country.

**Variable**	**Production quantity index**	**Pig inventory index**	**Export quantity index**	**Price for pigmeat**	**Ln of number of ASF outbreaks, domestic pigs**	**Ln of number of ASF outbreaks, wild boar**
Production quantity index	**1**	0.105	0.090	**−0.192**	0.112	−0.020
National pig inventory index	*0.201*	**1**	**0.413**	**−0.202**	**0.176**	0.059
Export quantity index	*0.274*	<*0.001*	**1**	**−0.262**	**0.308**	**0.353**
Price for pigmeat	*0.019*	*0.013*	<*0.001*	**1**	**−0.542**	**−0.623**
*Ln* of number of ASF outbreaks, domestic pigs	*0.174*	*0.032*	<*0.001*	<*0.001*	**1**	**0.725**
*Ln* of number of ASF outbreaks, wild boar	*0.813*	*0.472*	<*0.001*	<*0.001*	<*0.001*	**1**

*Correlation coefficient in bold are statistically significant at 5% risk level*.

### Estimation Results

According to the estimation results, the models explained altogether about 71% of the variation in the system of equations. The coefficient of determination for the price of the pigmeat equation was 85%. For other equations, this ranged from 43 to 48%. The error terms of four equations were correlated and these correlations were statistically significant. The cross-equation correlation was negative between the price of pigmeat and the export quantity equations. Other cross-equation correlations were positive. The largest cross-equation correlations were observed between the export quantity and the production quantity equation and the export quantity and the price equation.

**Figure 3:**
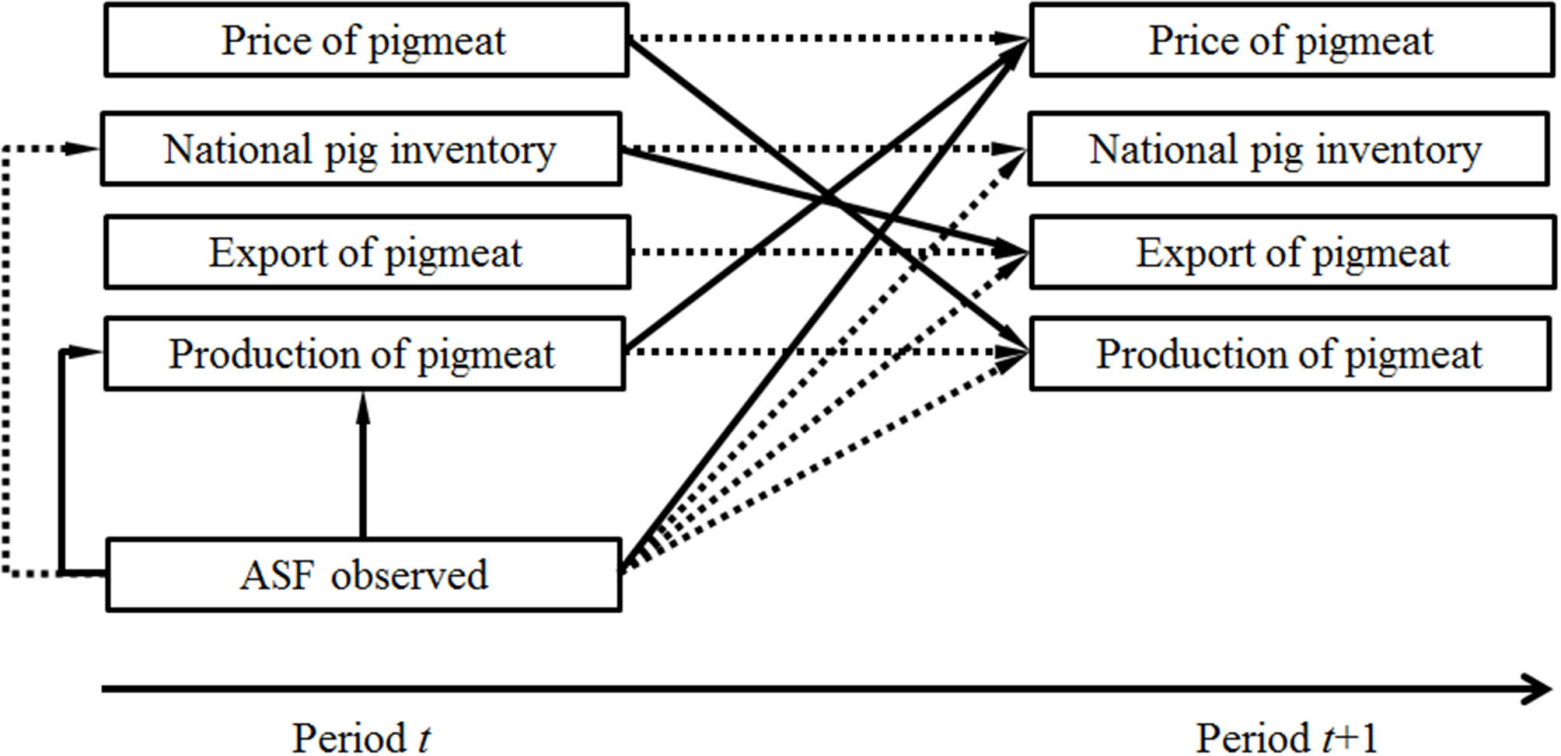
An illustration of the main effects of variables (presented in boxes) according to the estimated system of equations in two time periods [*t* (on the left) and *t*+1 (on the right)]. Dashed lines represent a negative association (an increase in one variable is associated with a decrease in another variable) and solid lines represent a positive association (an increase in one variable is associated with an increase in another variable). Observed cases of ASF in domestic pigs or wild boar are associated with variables in four uppermost boxes in the present period *t*. These all may be associated with variables on the right-hand in the next period (*t*+1).

An increase in the number of farms with ASF infection in a given year was associated with a decreased pig inventory and an increased production quantity in the same year. Moreover, this was associated with a decrease in both production quantity and exports in the next year. This observation was in line with the observations made from the raw data. An increase in the number of ASF outbreaks in wild boar in a given year was associated with an increase in the price of pig meat and a decrease in the national pig inventory in the next year (Table 4, Figure 3).

**Table 4:** Estimation results for a system of four simultaneous equations describing annual change in the logarithm of the national pig inventory, quantity of pigmeat produced, quantity of pigmeat exported and the price of pigmeat (class E).

	**Production quantity**		**Price of pigmeat**		**Pig inventory**		**Export quantity**	
	**Estimate**	***p*-value**	**Estimate**	***p*-value**	**Estimate**	***p*-value**	**Estimate**	***p*-value**
Intercept			1.519	< 0.001	3.215	< 0.001		
Dummy variable for year 2012	−0.068	0.0005	0.049	0.001				
Dummy variable for year 2013	−0.062	0.0028						
Dummy variable for year 2014	−0.094	< 0.001	−0.086	< 0.001				
Dummy variable for year 2015			−0.183	< 0.001				
Dummy variable for year 2016			−0.073	< 0.001				
Dummy variable for year 2018			−0.187	< 0.001				
Dummy variable for Bulgaria	0.133	< 0.001	0.047	0.018			0.218	0.009
Dummy variable for the Czech Republic	−0.142	< 0.001	0.056	0.002	−0.104	< 0.001		
Dummy variable for Estonia	0.067	0.007					0.264	0.002
Dummy variable for Italy	−0.112	< 0.001	0.060	0.002			0.228	0.025
Dummy variable for Latvia	0.127	< 0.001					0.275	0.002
Dummy variable for Lithuania					−0.118	< 0.001	0.285	0.001
Dummy variable for Hungary	−0.066	0.007	0.059	0.001				
Dummy variable for Poland					−0.086	< 0.001	0.397	< 0.001
Dummy variable for Romania	0.070	0.005					0.800	< 0.001
Dummy variable for Slovakia	−0.182	< 0.001	0.090	< 0.001	−0.076	0.001	0.358	< 0.001
*ln*(Price of pigmeat, *t–*1)	0.356	< 0.001	−0.400	< 0.001				
*ln*(Index of supplied quantity, *t–*1)	−0.374	< 0.001	0.114	0.001	−0.126	< 0.001		
*ln*(Index of national pig inventory, *t–*1)					−0.579	< 0.001	0.447	< 0.001
*ln*(Index of quantity of exports, *t–*1)							−0.442	< 0.001
*ln*(1+number of new ASF infected farms in *t–*1)	−0.018	0.021					−0.061	0.001
*ln*(1+number of new ASF infected wild boars in *t–*1)			0.0061	0.003	−0.007	0.009		
*ln*(1+number of new ASF infected farms in *t*)	0.016	0.009			−0.011	0.003		
Equation system *R*^2^	0.706							
Equation *R*^2^	0.476		0.854		0.430		0.428	
Equation *R*^2^ adjusted	0.389		0.832		0.379		0.363	
	**Production quantity**		**Price index**		**Number of pigs**		**Export quantity**	
**Cross-equation correlations**
Production quantity	1.000		0.095		0.011		0.424	
Price index of pigmeat	0.095		0.019		0.019		−0.189	
Number of pigs in the country	0.011		0.019		1.000		0.043	
Export quantity of pigmeat	0.424		−0.189		0.043		1.000	
	**Production quantity**		**Price index**		**Number of pigs**		**Export quantity**	
**Cross-equation significance estimates**
Production quantity	0.004		0.000		0.000		0.005	
Price index of pigmeat	0.000		0.002		0.000		−0.002	
Number of pigs in the country	0.000		0.000		0.002		0.000	
Export quantity of pigmeat	0.005		−0.002		0.000		0.044	

Some of the lagged variables representing the national pig inventory, quantity of pigmeat produced, quantity of pigmeat exported, or the price of pigmeat contributed to year-to-year changes in these variables. An increase in the producer price of pigmeat in a given year was related to a decrease in the price of pigmeat and to a decrease in the quantity supplied in the next year. An increase in the production quantity in a given year was related to a lowered production quantity and national pig inventory in the next year, and also to an increase in the producer price of pigmeat in the next year. An increase in the national inventory in the current year was associated with a decrease in the national pig inventory in the next year and an increase in export quantity in the next year. Finally, an increase in the exports of the current year was associated with a decrease in the quantity of exports in the next year (Table 4, Figure 3).

Several dummy variables representing years were significant in explaining any annual changes in the price of pigmeat and production quantity. Country-specific dummy variables were statistically significant especially in equations which represented production and export quantities.

## Discussion

The results indicate that the consequences of ASF to pigmeat markets in a country where ASF has been introduced are complex and confounded by possible interrelated and country-specific factors. Moreover, the estimation results suggest that the pigmeat markets can respond differently to the introduction of ASF into the country. The results suggest that on average, when taking into account the size of an outbreak, the new cases of ASF reduced pigmeat exports by close to 15% in the year after the cases had occurred, production quantity by more than 4%, and national pig inventory by 3–4% both in the current and the next year. The larger impact on exports is in line with the literature [e.g., (9–11, 30)].

The magnitude of impacts is affected by the size of epidemic. The larger and the more widespread the disease is, the larger can its impact expected to be. This is logical because also the policy measures (7) to combat ASF are context-specific. Stochastic simulation models [e.g., (10, 19)] have shown that the market effects of ASF, which are comprised of changes in prices and quantities traded, can vary substantially from case to case. For instance, Halasa et al. (19) found in a simulation carried out for Denmark that export losses caused by ASF varied between €250 and €383 million per epidemic. These studies also suggest that export losses play a larger role in the total losses than direct costs associated with disease control measures.

As it has been illustrated in previous studies for different diseases [e.g. (31)], an outbreak of a disease such as ASF can lead to a supply shock and a demand shock. The latter is usually associated with decreasing exports when countries prohibit the imports of pig products originating from the region or country where ASF occurs. In practice, the effects of these shocks depend on factors such as the size and duration of epidemic and reactions of trade partners. While changes in export quantities estimated in the present study were larger than those of production or prices, it is to be noticed that the market losses are comprised of the net effect of changes in both the price and quantity traded and these changes can take place over a longer time period. Another factor which may influence the magnitude of market losses suffered by the pig sector is disease control policy. For instance, Halasa et al. (9) found that increasing testing of dead animals in the protection and surveillance zones reduced both the duration of epidemic and economic losses caused by ASF.

This study attempted to assess the magnitude of the market components of outbreak by using data from actual ASF epidemics. The results suggest that an ASF outbreak can reduce the production of pigmeat, export quantities, and the national pig inventory (i.e., “production capacity”) in the short and medium term. Particularly, the decrease in the national pig inventory can be expected because of disease control and eradication measures. However, the production of pigmeat shortly after observing ASF in a country can even increase as a consequence of an ASF outbreak. One possible explanation for this is that farmers may perceive business prospects becoming less favorable and therefore they may begin culling animals, which may lead to slightly increased supply in the very short run and a reduced pig inventory. Moreover, restrictions imposed on farms in the protection and surveillance zones may raise slaughter weights and thus increase the supply locally after the restrictions are removed. Changes in the supply, national pig inventory, and exports of pigmeat can be expected to occur with a delay when the disease starts influencing the production capacity and export markets.

Although the price development over time is driven by global market developments, ASF outbreaks do impact local producer prices. A decrease in the producer price during the epidemic and an increase after the disease has been eradicated has been observed previously in simulation-based studies [e.g., (30, 32, 33)]. However, the present data did not suggest a substantial instantaneous drop in pigmeat price, which has been postulated in cases representing both ASF and other highly contagious animal diseases [e.g., (10, 14, 19, 34)]. This may be because of the EU policies to limit intra-community trade on a regional basis (7). The possibility for exceptional support measures (8) may also have relieved the impacts in the countries which were affected by ASF. In qualitative analysis, countries where ASF was introduced in 2014 did not face a development that was different from the other countries in the data. This may be related to the restrictions imposed by Russia on pigmeat of EU-origin, which—it has been argued—were an important reason for the decreasing price of pigmeat in the EU in 2014–2015. It may also be related to the afore-mentioned EU policies. Moreover, if the supply of meat in the region where a disease is present is reduced, then other regions which have not suffered from the disease can, in some cases, benefit from the outbreak, and these other regions may even increase their supply of pigmeat. Mangen and Burrell (30) have illustrated such a case at the national scale for classical swine fever, and Mason-D'Croz et al. (35) in the global context for ASF. Taking into account such differences within the country may smooth the effects at the country level. These aspects suggest that from the perspective of pig sector, it is essential to keep the restrictions on trade as limited as possible.

The country-specific price development appeared to be related to the EU-level price development. The development in Latvia may be associated with the fact that the price of pigmeat in the country was initially 20–38% lower than in any other member state included in the data in the same year. The dependent variables in the system of equations were in a first-differenced form, which together with the development of variables implied that there were significant country-level development trends in each of the four equations, and in addition, a general trend in the price and pig inventory equations (i.e., the intercept).

There are also other market considerations which are relevant to the market implications of ASF. For instance, it has been found that the farm gate price of beef in the UK decreased and retail price increased, and the average marketing margin of beef (retail price minus farm gate price) increased by 3.1% following the FMD outbreak when compared with the pre-FMD period (14). The present study did not consider the possibility that ASF would affect consumer preferences. Although this may be highly relevant in the event of some other diseases [such as zoonotic diseases or production diseases; see, e.g., (36) for discussion], in the event of ASF it is unlikely because ASF does not pose a risk to human health.

Meat markets may respond to disease events sluggishly and in different ways. The present result that the impact of the ASF variable on the price of pigmeat was positive can be related to reduced supply, which has been also observed in simulation-based studies. The results suggest that a reduced supply and production capacity (the national pig inventory) as a consequence of ASF can contribute to changes in pigmeat prices. Moreover, the markets may show stronger price impacts in the very short term than at an annual level, and these short-term impacts may be over-represented in public discussion. Regions where the disease has not been detected may also increase their supply and compensate for the loss of production in areas where the disease is present, as illustrated by previous studies (30, 35), and this may smooth out the markets effects of ASF at the country level.

Besides Europe, ASF was spreading in China and some other countries in Asia during 2018–2019. The epidemic in China, the largest pigmeat producer country in the world, has been estimated to impact both Chinese and global pigmeat markets. Although the effect of the Chinese epidemic on the pigmeat market was not considered explicitly in the present model, yearly dummy variables captured the overall effects of unspecified annual changes in the dependent variables, including the effects of ASF in Asia to the European pigmeat markets. However, such dummy variables cannot separate the effect of an individual event, such as ASF epidemic in China, from the effects of other unspecified events occurring in the same year. Mason-D'Croz et al. (35) projected that global pork prices could increase by 17–85% as a consequence of ASF epidemic in China. Recent EU agricultural markets outlook (37) also showed that the ASF situation in China will impact the price of pigmeat in the EU, and that the faster Chinese production will recover from ASF, the lower are prices in the EU and China forecast to be in the coming years.

The dynamics of supply, exports prices and pig inventory can play an important role in determining the impacts of ASF in the domestic pigmeat markets. While one can argue that the introduction of ASF into the country leads to a falling producer price of pigmeat, this may not be the full picture. ASF may lead to a decreasing pig inventory, supply and export of pig meat. This contributes to the balance between the supply and demand for pig meat in the domestic markets by decreasing the supply and subsequently exports may also decrease pigmeat. Hence, the market may not show large price reductions because reductions in supply can partly compensate for the effects of excess local supply in cases where some of the export markets become temporarily closed. In addition, export orientation of the country may also play a role in determining the impacts. Because EU policies can limit disruptions in the intra-community trade to only regions where the measures are limited (7), but third countries may apply the restrictions to the entire country, this may shift some pigmeat exports of an infected country from the third-country markets to the common market.

Caution must be taken when interpreting these results, because a reduced form model was estimated and the inclusion or exclusion of variables in the model may have an effect on the result. Including additional structures in further analyses to explain the market developments could clarify the results. These results also suggested that there are important country-specific trends, which must be controlled properly in the estimation. In addition, the effects of wider market shocks, such as the Russian embargo on importing pig products from the EU and a generic fall in the producer price of pigmeat, which may be a confounding factor in European markets in 2014–2015, and 2018–2019 events in the global pigmeat markets (especially ASF in China), must be controlled. In the current study they were taken into account by annual dummy variables. The ASF-affected time period available at the time of the study covered only a few years and not all effects may have been observable during the study period. Topics for further research, which were not examined in the present analysis, could conclude impacts of diseases on the exports of meat preparations such as ready-to-eat meals and regional differences within countries where a highly contagious animal disease has been observed.

## Conclusion

In conclusion, an ASF outbreak can influence the pigmeat markets adversely and these effects vary from country to country. An outbreak reduces the supply of pigmeat, exports and national pig inventory in the short term or in the longer term. The effects on pigmeat exports are likely to be stronger than the effects on production and prices, and the main effects may occur with a delay after the meat industry has used up the capacity to adjust the supply. The effects of ASF on market prices are complex, and decreasing supplies and exports can relax supply side pressures on the markets. The market effects of an ASF outbreak on the pig production sector is a combination of changes in prices, supply and trade, and these effects can change over time. When permitted by epidemiological situation, stakeholders are encouraged to promote the flexibility of the markets by limiting the market disruptions to the minimum, because flexibility of trade can help to reroute trade flows and mitigate the negative effects of ASF to the pig sector.

## Data Availability Statement

The datasets for this study can be found in the websites of Eurostat and European commission (see references), or are available from the author upon request.

## Author Contributions

The author confirms being the sole contributor of this work and has approved it for publication.

## Conflict of Interest

The author declares that the research was conducted in the absence of any commercial or financial relationships that could be construed as a potential conflict of interest.
